# Morbidity status and changes in difficulty in activities of daily living among older adults in India: A panel data analysis

**DOI:** 10.1371/journal.pone.0269388

**Published:** 2022-06-02

**Authors:** Priyanka Patel, T. Muhammad, Harihar Sahoo

**Affiliations:** Department of Family and Generations, International Institute for Population Sciences, Mumbai, Maharashtra, India; University of Copenhagen: Kobenhavns Universitet, DENMARK

## Abstract

**Introduction:**

The study explored the socioeconomic and demographic factors that determine the onset of difficulty, recovery from difficulty and difficulty remaining in functional activity in later years of life. Additionally, the study examined the effects of several combinations of chronic diseases on the changes in later-life functional difficulty.

**Methods:**

We used data from two rounds of India Human Development Survey (IHDS) conducted during 2004–2005 and 2011–2012. A sample of 13,849 respondents aged 55 years and above with a seven year follow-up was considered for this study. The Katz Index of Independence in activities of daily living (ADL) was used to measure the functional disability as an outcome variable. Multinomial logistic regression has been conducted to fulfil the study objectives.

**Results:**

The overall functional difficulty among older adults was 27.3% and onset of functional difficulty (23.5%) was higher than the recovery from difficulty (2.1%) and remaining with difficulty (1.7%). Onset of functional difficulty in second round was higher among women (27.3%) than men (19.3%). Bivariate and multivariate analyses showed that single and multi-morbidity had a positive significant association with all categories of functional difficulty. Female sex, increasing age and rural place of residence had positive association with onset of difficulty and difficulty remaining in second round. The combinations of morbidities were also found to have positive significant association with functional difficulty i.e., the relative risk (RR) of onset of difficulty in second round is higher among those who had diabetes with high blood pressure (RR-1.7; CI: 1.4–2.0), cataracts with high blood pressure (RR-2.0; CI: 1.5–2.6) and cataracts with asthma (RR-3.1; CI: 2.1–4.6) compared to those with no diabetes and cataract but with high blood pressure or asthma, respectively.

**Conclusion:**

The findings suggest that the risk of onset of functional difficulty is higher among older individuals with single and multiple morbidities compared to their healthy counterparts. It is also found that functional difficulty increased with age and was more prevalent in older women and rural residents, suggesting the need for appropriate policy interventions with special focus on the vulnerable senior adults.

## Introduction

The world continues to see an extraordinary and long-term shift in the age structure of the global population, owing to rising life expectancy. People are living longer lives, and the percentage and number of older people in the population are steadily increasing [1, 2]. In the 21st century, population ageing is the major demographic issue in India with enormous consequences for the economy and society. It is almost inevitable that India will experience a transition from a "young country" to an "elder country" in the next decades due to fast changes in the demographic indicators. In 2011, 8.6% of the population (104 million) was over the age of 65. By 2050, India’s older age population is projected to be at 19% (around 300 million) [3]. Around 5% of the older population in India are affected by some kind of physical limitation [4], and the burden of disease is predicted to increase substantially due to rising life expectancy and associated population aging.

Previous research has shown that there is a strong association between chronic morbidities and functional limitation [5, 6]. As severe morbidity disrupts normal daily activities, it also reduces quality of life [7–10]. Some studies have found that arthritis [6, 11, 12], cardiovascular disease, lung disorders, vision disabilities, and diabetes [12, 13], are common causes of functional limitation in older ages. The interaction of various chronic diseases with functional limitation [14] and the combined effects of two or more diseases [15] have also been documented in previous studies. Chronic diseases are associated with increased rate of disability, reduced functional levels, increased poly-pharmacy, poor health-related quality of life (HRQoL) and more health care needs [15–20]. The impact of multimorbidity on individuals’ health profiles surpasses the impact we would expect from the summed effect of single conditions [21]. Multimorbidity leads to physical decline, and people with more conditions, more severe disease and specific disease patterns experience steeper deterioration [22].

Older individuals with multimorbidity are at greater risk for disability, hospitalization, postoperative complications, and mortality [23, 24]. Longitudinal studies have shown that multimorbid older individuals have poorer quality of life and inferior functional capacity or reduced physical functioning [25, 26], and almost half of those living with any chronic conditions also suffer from some activity limitations [27]. Limitations in physical and cognitive functions due to multimorbidity decisively affect people’s illness and treatment burden and their response capacity, which may further increase multimorbidity [28, 29]. According to Kadam and Croft, 24% of the burden of poor physical functioning in the family practice may be attributable to higher prevalence of multimorbidity [30]. Multimorbidity and the severity of morbidities were associated with poor physical functioning [31]. Vancampfort *et al*. (2017) found that older age is highly associated with multimorbidity and low physical activity [32]. Most chronic conditions were associated with low physical activity in the overall sample, although this relationship was most notable among the older population in low and middle-income countries [33]. Furthermore, physical activity was consistently associated with better physical health and mental health in males and females and in young and older adults [34].

The etiological pathways from chronic diseases to limitations in activities of daily living or functional difficulties are documented in earlier studies [35, 36]. However, there is a dearth of studies that focus on association of chronic conditions with functional difficulties of older individuals in low and middle-income countries and in India in particular. The ones that do so only focus on cross-sectional associations [32, 37, 38]. The present study is aimed to explore how single and multi-morbidity status longitudinally affects functional difficulty among older adults. The study also explores the socioeconomic and demographic factors that determine the onset of functional difficulty, recovery from functional difficulty and remaining with functional difficulty in later years of life using panel data of older Indian adults. Additionally, the study examines the effects of several combinations of chronic diseases on the changes in late-life functional difficulty.

## Methods

### Data

In this study, we used data from two rounds of India Human Development Survey (IHDS) conducted jointly by the University of Maryland and National Council for Applied Economic Research (NCAER) during 2004–2005 and 2011–2012. The IHDS contains nationally representative multi-topic longitudinal panel data [39, 40]. The first round of IHDS (IHDS-I) (2004–05) covered 41,544 households in 1503 villages and 971 urban neighbourhoods while the second round of IHDS (IHDS-II) (2011–12) covered 42,152 households in 384 districts, 1420 villages and 1042 urban neighbourhoods. About 83 percent of the households interviewed in the first round (2004–05) were re-interviewed in the second round of IHDS [40]. The data is freely available in the public domain and survey agencies that conducted the field survey for the data collection have collected prior consent from the respondent. Therefore, prior ethical approval for using the datasets was not required. As the objective is to explore the determinants of changes in functional health among older population, both the survey rounds have been considered and a panel data have been created (merging of IHDS-I and IHDS-II) for this study.

**Fig 1:**
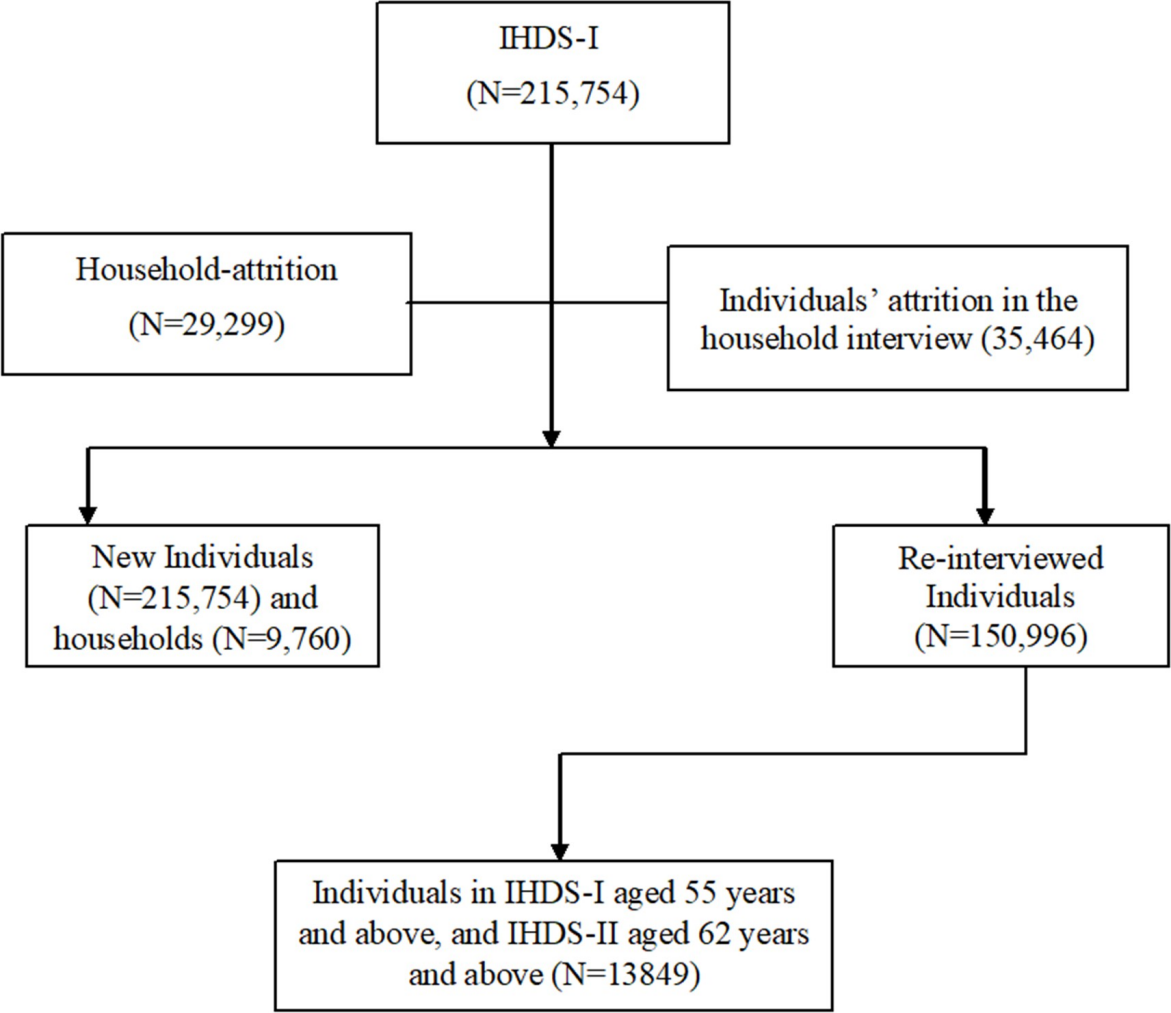
Flow chart of the sample selection in the study. Sources: The authors referred the IHDS website and the IHDS merging guide.

While merging the data, in IHDS-I, we use the sample of age group 55 years and above who were considered older adults in previous Indian studies [41, 42], and in IHDS-II, we use the follow-up older population aged 62 years and above after seven years. After combining the sample from IHDS-1 and IHDS-II, the new sample was 13,849 older individuals who were followed in both IHDS rounds. The sample selection criteria have been summarized in Fig 1. In the IHDS survey, information on morbidity and reported functional difficulty were recorded in the form of fifteen chronic morbidities and limitations in seven types of Activities of Daily Living (ADL). In this study, we define functional difficulty as “functional limitation” in the performance of ADLs among older individuals aged 62 years and above.

### Variable description

#### Dependent variable

In the present study, we considered a functional limitation in three ADLs among older persons: walking, toileting, and dressing. The question asked in the survey was: “Now, I am going to ask you about any physical difficulty that people above the age of 60 in this household may have. Does anyone in the household have a problem?” If the response is “yes,” the next question is: “Can (name of the affected person) still do it with some trouble or is he/she unable to do it?” with options for three types of ADL: “i) walking 1 km; ii) going to the toilet without help; iii) dressing without help”. All these options have three responses, namely, “No difficulty”, “Can do with difficulty” and “Unable to do it”. We computed the Katz index of independence in ADL, referred to as the ‘Kz score’, to assign coding to these categories. Hence, a Kz score = 0 signifies “Unable to do it”, a Kz score = 1 signifies “Can do with difficulty”, and a Kz score = 2 signifies “No difficulty”. The Katz Index of Independence in ADL, also called “Katz ADL” is the standardised index for measuring the index of functional difficulty in ADL [43]. We classify all types of functional difficulties in binary form, where, “1” represents “no difficulty or functional independence” and “0” represents functional dependence that includes both “can do it with difficulty” and “unable to do it”. The ’Kz score’ was created by adding the scores for all three forms of functional difficulties. Now, we create final dependent variable by using merged data (panel data) of both rounds (IHDS-I & IHDS-II). Here variable term "no difficulty in both round" refers to “those individuals who experienced no functional difficulties in both rounds”; "onset of difficulty in second round" refers to “those individuals who did not experience functional difficulties in IHDS-I but experienced functional difficulties in IHDS-II; "difficulty recovered in second round" refers to “those individuals who experienced functional difficulties in IHDS-I but not in IHDS-II”; and "functional difficulty remains in both the rounds" refers to “those individuals who experienced functional difficulties in both rounds”.

Final dependent variable was recoded to four categories as; i) no functional difficulty in both the rounds ii) onset of functional difficulty in second round iii) functional difficulty recovered in second round and iv), functional difficulty remains in both the rounds.

#### Exposure variables

The main exposure variable in our analysis was the presence of chronic morbidities. The exact question was: “Has a doctor ever diagnosed any member in the household as having–high BP/heart disease/diabetes/cancer/cataract/tuberculosis/asthma?” These morbidities were recoded as, ‘1’ that refers to “morbidities present” and ‘0’ that refers to “morbidities absent”. Following the available literature [44–46], we included a set of demographic and socioeconomic variables in the regression model. These are: age (62–69, 70–79, 80 and above); sex (male or female); headship (yes, no); tobacco chewing (ever, never); tobacco smoking (ever, never), alcohol consumption (ever, never); below poverty line (BPL) (yes, no) (assessed through the question, “does the household have a BPL card?” which captures the aspect of financial inclusion, unlike wealth index [47]); place of residence (rural or urban); marital status (currently married and other); religion (Hindu, Muslim and other); caste (Scheduled Caste (SC) / Scheduled Tribe (ST), Other Backward Class (OBC) and Others), wealth quintiles (poorer, poorest, middle, richer and richest); education (illiterate, primary, secondary and higher); morbidity (no morbidity, single and two and above morbidity) and region (north, south, east, west. northeast and central).

### Statistical analysis

First, we used descriptive statistics and bivariate analysis. Further, Chi-square test was used to show the level of significance in the possible associations between functional difficulty and background characteristics. Dyads pattern was identified by utilizing a basic matrix technique to conduct an exhaustive study of all conceivable combinations of two co-morbid conditions using a simple descriptive statistical method [48]. Dyads of chronic morbidities in the study include diabetes with cataract, heart disease, asthma and high blood pressure; cataract with heart disease, asthma and high blood pressure; heart disease with asthma and high blood pressure; and asthma with high blood pressure. To analyze the relationships between morbidity, socioeconomic characteristics and functional difficulties, we used multinomial logistic regression model.

Multinomial logistic regression equation is written as:

$$RR=\frac{P(Y=1|X+1)/P(Y=base\;category|X+1)}{P(Y=1|X)/P(Y=base\;category|X)}$$

The estimates of multinomial logistic regression are presented in the form of Relative Risk (RR) and P is the probability of occurrences in the equation.

Multinomial logistic regression was used with four categories of functional difficulties; i) no functional difficulty in both rounds ii) onset of functional difficulty in second round iii) functional difficulty recovered in second round and iv) functional difficulty in both rounds. No functional difficulty was considered as reference category.

## Results

Table 1 depicts the percentage distribution of panel data by the socioeconomic and demographic characteristics of the study participants. In the panel data, 47.2% of the participants belonged to 62–69 years, 37.8% to 70–79 years and 15% to 80 years and above.

**Table 1:** Distribution of older adults by their socio-economic and demographic characteristics.

Characteristics	Panel sample (n = 13,849)	
	n	%
**Age**		
62–69	6,534	47.2
70–79	5,235	37.8
80 and above	2,079	15.0
**Sex**		
Male	6,932	50.1
Female	6,917	50.0
**Headship**		
Yes	7,313	52.8
No	6,536	47.2
**Marital status**		
Currently married	7,992	58.3
Not currently married	5,716	41.7
**Education**		
No education	2,830	20.4
Primary	4,617	33.4
Secondary	3,637	26.3
Higher	2,761	19.9
**Smoking tobacco**		
Never	11,769	85.0
Ever	2,080	15.0
**Chewing tobacco**		
Never	10,640	76.8
Ever	3,209	23.2
**Consuming alcohol**		
Never	12,693	91.7
Ever	1,156	8.3
**Wealth Index**		
Poorest	3,519	25.4
Poorer	2,142	15.5
Middle	2,350	17.0
Richer	2,609	18.8
Richest	3,229	23.3
**BPL**		
Yes	2,853	20.6
No	10,996	79.4
**Religion**		
Hindu	11,642	84.1
Muslim	1,269	9.2
Others	938	6.8
**Caste**		
SC/ST	3,446	24.9
OBC	5,168	37.3
Others	5,235	37.8
**Place of residence**		
Rural	10,292	74.3
Urban	3,557	25.7
**Region**		
North	1,949	14.1
Central	2,928	21.1
East	2,938	21.2
North-East	297	2.1
West	2,242	16.2
South	3,494	25.2

Panel sample refers to the population who were alive in both rounds of IHDS; n: Un-weighted count, %: Weighted percentages to account for population estimates.

Table 2 presents the bivariate analysis showing the prevalence estimates of changes in functional difficulty from first round to second round among older adults in India, and it varies by background characteristics, which had four categories “no difficulty in both the rounds”, “onset of difficulty in second round”, “recovery from difficulty in second round” and “difficulty in both the rounds”. The onset of difficulty, difficulty recovery and difficulty remaining increased with increasing age. The onset of difficulty in the second round (27.3% vs 19.7%), difficulty recovery in the second round (2.3% vs 2.0%) and difficulty remaining in second round (2.2% vs 1.2%) were higher among females as compared to males. In case of geographical regions, southern region (28.6%) showed higher functional difficulty and north-east region (6.6%) had lower level of onset of functional difficulty in the second round.

**Table 2:** Prevalence of onset, recovery from and remaining with functional difficulty among older adults by background characteristics.

Characteristics	Change in functional difficulty status								
	Onset of functional difficulty			Recovered from functional difficulty			Remained with functional difficulty		
	N	%	p-value	N	%	p-value	N	%	p-value
**Morbidity**									
No	1,785	19.2	<0.001	162	1.7	<0.001	114	1.1	<0.001
Single morbidity	944	29.6		93	2.8		78	2.2	
Multi-morbidity	576	37.7		56	3.6		79	4.9	
**Age**									
62–69	1,038	15.9	<0.001	90	1.4	<0.001	72	1.1	<0.001
70–79	1,361	26.0		145	2.8		86	1.6	
80 and above	851	40.9		62	3.0		80	3.9	
**Sex**									
Male	1,359	19.7	<0.001	139	2.0	<0.001	93	1.2	<0.001
Female	1,946	27.3		172	2.3		178	2.2	
**Headship**									
Yes	1,440	20.7	<0.001	143	2.1	0.036	93	1.5	<0.001
No	1,865	27.3		168	2.2		178	2.1	
**Marital status**									
Currently married	1,582	19.4	<0.001	154	1.8	0.002	107	1.2	0.001
Not currently married	1,693	29.2		155	2.7		161	2.5	
**Education**									
No education	622	24.9	<0.001	60	2.2	0.031	35	1.4	0.208
Primary	1,149	24.5		92	2.0		98	1.8	
Secondary	929	23.3		108	2.5		82	2.0	
Higher	604	20.5		50	1.8		56	1.6	
**Smoking tobacco**									
Never	2,879	24.1	<0.001	272	2.2	0.180	237	1.8	0.212
Ever	426	20.1		39	2.0		34	1.3	
**Chewing tobacco**									
Never	2659	23.9	0.500	265	2.3	0.022	232	1.9	0.021
Ever	646	22.1		46	1.7		39	1.1	
**Consuming alcohol**									
Never	3,095	24.0	<0.001	285	2.1	0.862	258	1.8	0.044
Ever	210	18.1		26	2.5		13	1.1	
**Wealth Index**									
Poorest	803	25.3	<0.001	67	2.1	0.534	66	2.0	0.550
Poorer	476	24.6		45	2.0		33	1.2	
Middle	544	22.0		44	1.8		48	1.8	
Richer	638	22.3		75	2.5		57	2.0	
Richest	844	22.8		80	2.2		67	1.4	
**BPL**									
Yes	688	24.7	0.006	55	1.9	0.503	60	2.1	0.209
No	2,617	23.2		256	2.2		211	1.6	
**Religion**									
Hindu	2,736	23.5	0.436	245	2.1	0.133	207	1.6	<0.001
Muslim	304	22.2		35	2.6		25	1.7	
Others	265	25.3		31	2.6		39	3.8	
**Caste**									
SC/ST	736	21.9	<0.001	67	1.9	0.450	50	1.6	0.086
OBC	1,283	26.1		107	2.2		98	1.7	
Others	1,286	22.0		137	2.3		123	1.8	
**Place of residence**									
Rural	2,475	24.0	0.002	227	2.1	0.925	214	1.9	0.020
Urban	830	21.8		84	2.3		57	1.3	
**Region**									
North	770	24.6	<0.001	75	2.4	<0.001	50	1.4	<0.001
Central	741	25.7		29	1.0		45	1.3	
East	384	19.9		59	2.7		22	1.1	
North-East	19	6.6		1	0.2		1	0.4	
West	359	18.5		41	1.9		22	1.2	
South	1,032	28.6		106	2.8		131	3.3	
**Total**	**3,305**	**23.5**	** **	**311**	**2.1**		**271**	**1.7**	

N: Un-weighted counts, %: Weighted percentages to account for population estimates; p-values are based on Chi-square test.

Table 3 reveals the correlates of onset, recovery and remaining with functional difficulty among older adults. Older participants with single morbidity were 1.9 times more likely to have onset of difficulty, 2 times more likely to have difficulty recovered and 2.5 times more likely to have difficulty remained, compared to those with no morbidity. Older adults who had multi-morbidity were 3.3 times more likely to have onset of difficulty, 3 times more likely to have difficulty recovered and 6 times more likely to have difficulty remained, compared to those with no morbidity.

**Table 3:** Relative risk ratio from multinomial logistic regression showing the association of multi-morbidity with onset, recovery from and remaining with functional difficulty among older adults (ref: No functional difficulty in both rounds) by background characteristics.

Characteristics	Change in functional difficulty status		
	Onset of functional difficulty	Recovered from functional difficulty	Remained with functional difficulty
	RR (95% CI)	RR (95% CI)	RR (95% CI)
**Morbidity**			
No®			
Single morbidity	1.9***(1.76 2.15)	2.0***(1.55 2.63)	2.5***(1.87 3.42)
Multi-morbidity	3.3***(2.87 3.74)	3.0***(2.16 4.23)	6.0***(4.33 8.36)
**Age**			
62–69®			
70–79	1.8***(1.66 2.01)	2.0***(1.53 2.63)	1.9***(1.38 2.58)
80 and above	3.8***(3.35 4.29)	3.5***(2.52 4.93)	5.7***(4.04 8.03)
**Sex**			
Male®			
Female	1.4***(1.2 1.57)	1.0 (0.72 1.49)	1.7***(1.16 2.58)
**Head**			
Yes®			
No	1.1 (0.97 1.22)	1.3 (0.95 1.76)	1.2 (0.85 1.64)
**Marital status**			
Not currently married ®			
Currently married	1.3***(1.13 1.38)	1.3**(1.01 1.74)	1.5***(1.14 2.09)
**Education**			
No education®			
Primary	1.0 (0.89 1.14)	0.8 (0.54 1.09)	1.6**(1.02 2.38)
Secondary	0.9 (0.79 1.05)	0.9 (0.63 1.32)	1.4 (0.9 2.25)
Higher	0.8**(0.69 0.97)	0.6**(0.36 0.91)	1.5 (0.89 2.52)
**Smoking tobacco**			
Ever®			
Never	1.0 (0.88 1.13)	0.9 (0.66 1.29)	0.7*(0.46 1.01)
**Chewing tobacco**			
Ever®			
Never	0.9 (0.83 1.03)	1.1 (0.84 1.51)	1.2 (0.88 1.71)
**Consuming alcohol**			
Ever®			
Never	1.1 (0.91 1.24)	0.9 (0.63 1.43)	1.4 (0.8 2.33)
**Wealth Index**			
Poorest®			
Poorer	0.9 (0.78 1.04)	1.0 (0.68 1.5)	0.7*(0.43 1.05)
Middle	0.9**(0.74 0.98)	0.8 (0.53 1.19)	0.8 (0.51 1.14)
Richer	0.8**(0.73 0.96)	1.1 (0.76 1.6)	0.7*(0.48 1.06)
Richest	0.8***(0.7 0.93)	0.8 (0.57 1.25)	0.6***(0.37 0.84)
**BPL**			
No®			
Yes	1.1*(0.99 1.24)	1.0 (0.75 1.41)	1.5***(1.12 2.13)
**Religion**			
Hindu®			
Muslim	1.0 (0.82 1.14)	1.2 (0.77 1.75)	0.9 (0.56 1.47)
Others	1.2**(1.03 1.45)	1.4 (0.91 2.1)	1.9***(1.29 2.91)
**Caste**			
SC/ST®			
OBC	1.1**(1 1.26)	1.1 (0.78 1.49)	1.2 (0.8 1.66)
Others	1.0 (0.89 1.14)	1.1 (0.79 1.55)	1.3 (0.9 1.92)
**Place of residence**			
Urban®			
Rural	1.2***(1.06 1.31)	1.2 (0.88 1.55)	1.9***(1.35 2.57)
**Region**			
North-East®			
North	1.3***(1.14 1.49)	0.6**(0.37 0.93)	1.4 (0.9 2.2)
Central	0.7***(0.56 0.77)	1.2 (0.83 1.77)	0.7 (0.42 1.23)
East	0.2***(0.11 0.29)	0.1**(0.02 0.81)	0.2*(0.02 1.27)
West	0.7***(0.57 0.77)	0.9 (0.57 1.28)	0.7 (0.43 1.22)
South	1.3***(1.15 1.46)	1.5**(1.07 2.04)	2.6***(1.84 3.79)

®: Reference category

* if p<0.10

** if p<0.05

*** if p<0.01, RR: Relative Risk, CI: Confidence Interval.

Table 4 presents the percentage distribution, bivariate and multivariable estimates of change in functional difficulty status by morbidity combinations during both the rounds. Having no functional difficulty in both rounds was taken as the base category, and having no morbidity combination was taken as the reference category for each morbidity combination. Participants with combination of diabetes and cataract were 1.6 times (RR: 1.6, CI: 1.1, 2.4) more likely to have onset of functional difficulty than those who had no morbidity combination. Older adults with diabetes and heart disease were 3.2 times (RR: 3.2, CI: 1.3, 7.9) more likely to be remain with functional difficulty in second round. Older adults with diabetes and asthma were 1.7 times (RR: 1.7, CI: 1.0, 30.) more likely to have onset of difficulty than their counterparts. Respondents with diabetes and high blood pressure were 1.7 times (RR: 1.7, CI: 1.4, 2) more likely to have onset of difficulty, 1.9 times (RR: 1.9, CI: 1.1, 3) more likely to have recovered from functional difficulty and 3.8 times (RR: 3.8, CI: 2.5, 5.8) more likely to remain with difficulty than those who had no morbidity combination. Older individuals with heart disease and high blood pressure were 1.4 times (RR: 1.4, CI: 1, 2) more likely to have onset of functional difficulty compared to those who had no morbidity combination.

**Table 4:** Percentage distribution, bivariate and multivariable estimates of changes in functional difficulty (ref: No functional difficulty in both rounds) by morbidity dyads.

Morbidity Combination	N (%)	Functional difficulty					
		Onset of difficulty (%)	RR (95% CI)	Recovery from difficulty (%)	RR (95% CI)	Remained with difficulty (%)	RR (95% CI)
No morbidity combination ®							
DI+CA	199 (1.44)	49.0	1.6*** (1.1 2.4)	4.7	1.7 (0.7 4.1)	5.4	1.0 (0.4 2.4)
DI+HD	174 (1.26)	41.8	1.3 (0.8 1.9)	3.4	1.8 (0.6 5.2)	7.7	3.2** (1.3 7.9)
DI+ASTH	80 (0.58)	50.4	1.7* (1.0 3.0)	4.3	2.7 (0.5 13.2)	10.2	1.5 (0.5 4.9)
DI+HBP	660 (4.77)	38.2	1.7*** (1.4 2.0)	4.1	1.9** (1.1 3.0)	6.6	3.8*** (2.5 5.8)
CA+HD	105 (0.76)	38.6	0.8 (0.5 1.4)	1.9	0.4 (0.1 1.9)	5.5	0.7 (0.2 2.3)
CA+ASTH	163 (1.18)	42.6	3.1*** (2.1 4.6)	2.4	2.0 (0.6 7.0)	7.5	4.2*** (1.8 9.8)
CA+HBP	308 (2.23)	44.5	2*** (1.5 2.6)	5.0	3.1*** (1.6 6.0)	6.1	3.7*** (1.9 7.3)
HD+ASTH	79 (0.57)	37.4	1.3 (0.7 2.4)	0.0	1.1 (0.8 3.5)	9.1	2.9* (0.9 8.8)
HD+HBP	272 (1.96)	36.8	1.4** (1.0 2.0)	2.9	1.1 (0.4 3.1)	5.5	0.7 (0.2 1.9)
ASTH+HBP	173 (1.25)	38.9	1.2 (0.8 1.7)	0.3	0.2 (0.0 1.6)	8.2	1.4 (0.5 3.7)

N: Un-weighted counts, %: Weighted percentages to account for population estimates, ®: Reference category

* if p<0.10

** if p<0.05

*** if p<0.01, RR: Relative Risk, CI: Confidence Interval, DI- Diabetes, CA- Cataract, HD-Heat Disease, ASTH- Asthma, HBP- High Blood Pressure.

## Discussion

This study aimed to find the prevalence of functional difficulty and explore the determinants of onset, recovery from and remaining with functional difficulty among older population using a panel data in India. Our study found that there is a substantial increase in the onset of functional difficulty with age i.e., 16 percent in the age group 62–69 to 41 percent in the age group 80 and above. The finding corroborates with the findings of a previous study in India that reported that 63% of older adults at the baseline and 67.3% of them at the follow-up after two year had at least one ADL limitation [49]. Similarly, functional difficulty in ADL and instrumental ADL is most frequently reported in the age group of 65 years and over than the age group of 40–64 years [6]. Another important finding of our study is the higher onset of disability among older women compared to men. This is in line with earlier studies in developed countries that have documented that, women have greater prevalence of impairment in most of the health outcomes [12, 50–52]. Recent population-based studies in India also reported that older women rate their health as poor more often than older men and have higher rates of functional difficulties [37, 53]. Gender disparity in disability is also explained by the fact that women live longer than men and therefore they are more likely to age with disability [54, 55].

The present study revealed higher chances of disability among the respondents with single or multiple morbidities in comparison to people with no morbidity. Concordantly, a previous study in a community-setting in Canada has shown that people with adverse health condition and comorbidity were 19 times more likely to have moderate to severe disability than those without such conditions [56]. Various longitudinal and cohort analyses have also found the negative influence of adverse health behaviors and morbidity on functional health among older individuals [57–59]. Notably in India, as people age, disability becomes a significant concern, increasing the caregiving burden for those who have to care for them [1]. On the other hand, a study shows that the old-age disability decreases with a lower rate of chronic morbidity [7]. Hence, individual disease-based interventions are required to ensure active aging among older Indian adults. Another finding of the current study showed that recovery from functional difficulty is higher among older adults with multi-morbidity. This finding can be explained by the higher attrition rate in the IHDS survey among urban residents (about 26%) than rural counterparts (about 9%) [60] and studies showed the differential morbidity and mortality pattern across socioeconomic groups in adulthood [61, 62]. The finding can also be attributed to the low sample size in the multi-morbid category and lack of statistical power in the current analysis. Similarly, the methodological shortcoming of the multinomial logistic regression leading to biased coefficient estimates when the sample size is small is acknowledged in previous studies [63]. Therefore, this needs to be further investigated with large sample size and more information from future longitudinal datasets.

Another important finding of the present study is the association of multimorbidity with functional difficulty. We had considered 7 diseases for calculating multimorbidity. The older adults in our study suffering from both cataract and asthma or diabetes and high blood pressure were more likely to have functional difficulty (either the onset of functional difficulty or to remain with functional difficulty in both the rounds of the survey). The study corroborates with earlier studies reporting three way combinations of chronic conditions such as hypertension, heart disease and arthritis [48]. The findings are also in line with a study that demonstrated the most common pairing of hypertension and diabetes with arthritis [64]. The same study revealed the presence of arthritis and chronic pain in most of the disease triads, quartets and quintets. It is suggested that any acute or sub-acute condition can affect the management of other diseases, for instance, leg injury of individuals can affect their mobility which can negatively affect their diabetes control and increase the chances of osteoarthritis [65].

Further, in the present study, functional difficulty is observed higher among currently unmarried respondents including widows. This supports the notion that in India where women have traditionally depended on the spouses for support, widowhood may have considerable negative impact on their socioeconomic and health status [41, 66]. The older people from the poorest and BPL category had a higher onset of functional difficulty compared to the richest and non-BPL population. Their lower functional health may be a result of their lower-income and lack of resources especially when they have higher levels of morbidity [18]. This is also supported by a positive poverty-disability association as reported in a systematic review including 150 studies in low- and middle-income countries [67]. A recent study found that the economically better-off older population experienced a lower prevalence of functional difficulty [46]. Supporting this, the disability among older adults in rural areas and those with socioeconomically poor backgrounds was higher than among urban resident and high socioeconomic groups, which is also similar to previous findings [4]. Another study conducted among 750 older individuals aged 60 years and above in Tamil Nadu, India, showed that physical disability was higher in rural areas [68]. Similarly, older people from southern region of the current study were more likely to have functional difficulty than those from other regions of the country, suggesting the need for further investigation of regional variations in the trajectory of functional difficulty.

There are several limitations of the study to be acknowledged. Because of the smaller sample size, we could not include the individual impact of significant morbidities on functional limitation. It would have been better for policy recommendations if the sample size had been large enough to independently analyse each disease and associated functional difficulty. Also, self-reporting and proxy reporting were possible in some situations, while there were differences in older adults’ health state in multiple places. However, the literature on the direction of proxy respondents’ reporting (either under-reporting or over-reporting) is not convincing [69]. Data from the IHDS share information on the disability conditions of the older population as reported by women (aged 15–49) in homes that meet the study requirements. The absence of information of disability directly from respondents rather than proxy respondents might lead to several biases and can be a drawback of this study. Notwithstanding these, the panel data which provide comprehensive information of an older cohort in two different time periods is the major strength of the study. Also, the combinations of different diseases were possible with multiple options, which adds to the present study’s credibility.

## Conclusion

This study provides information on morbidity status and associated changes in functional difficulties among older population in India. It is clearly evident that the single and multimorbidity status affects functional difficulty among older adults. The findings suggest that the likelihood of onset of functional difficulty is higher in older population with single and multiple morbidities compared to their healthy counterparts. It is also found that functional difficulty increased with age and was highly prevalent in older women compared to men. Higher levels of education and household wealth are revealed to be reducing functional difficulty and increasing the recovery rate. The current finding of the higher recovery from functional difficulty among older adults with multimorbidity requires further investigation with future longitudinal datasets. Marital status, religion, and place of residence also determined the changes in functional difficulty in the present study, suggesting the need for appropriate policy interventions with special focus on older women and oldest old adults.

## Abbreviations

RR: Relative Risk
CI: Confidence Interval
IHDS: India Human Development Survey
OBC: Other Backward Classes
SC: Scheduled Caste
ST: Scheduled Tribe

## References

[1] UNFPA. Caring for Our Elders: Early Responses India Ageing Report-2017. New Delhi, India, 2017.

[2] World Population Ageing 2019. 64.

[3] United Nations. World population prospects 2019, https://population.un.org/wpp/Download/Standard/Population/ (2019).

[4] Velayutham B, Kangusamy B, Joshua V, et al. The prevalence of disability in elderly in India–Analysis of 2011 census data. *Disabil Health J* 2016; 9: 584–592. doi: 10.1016/j.dhjo.2016.04.003 27174073

[5] Fried LP, Bandeen-Roche K, Kasper JD, et al. Association of comorbidity with disability in older women: The Women’s Health and Aging Study. *J Clin Epidemiol* 1999; 52: 27–37. doi: 10.1016/s0895-4356(98)00124-3 9973071

[6] Martin LG, Schoeni RF. Trends in Disability and Related Chronic Conditions Among the Forty-and-Over Population: 1997–2010. *Disabil Health J* 2014; 7: 1–23. 24456683 10.1016/j.dhjo.2013.06.007 PMC4151570

[7] Freedman VA, Martin LG. Contribution of chronic conditions to aggregate changes in old-age functioning. *Am J Public Health* 2000; 90: 1755–1760. doi: 10.2105/ajph.90.11.1755 11076245 PMC1446390

[8] Liang J, Liu X, Gu S. Transitions in functional status among older people in Wuhan, China: Socioeconomic differentials. *J Clin Epidemiol* 2001; 54: 1126–1138. doi: 10.1016/s0895-4356(01)00390-0 11675164

[9] Costa DL. Changing chronic disease rates and long-term declines in functional limitation among older men. *Demography* 2002; 39: 119–137. doi: 10.1353/dem.2002.0003 11852833

[10] Tey NP, Lai SL, Teh JKL. The debilitating effects of chronic diseases among the oldest old in China. *Maturitas* 2016; 94: 39–45. doi: 10.1016/j.maturitas.2016.08.016 27823743

[11] Martin LG, Freedman VA, Schoeni RF, et al. Trends in disability and related chronic conditions among people ages fifty to sixty-four. *Health Aff (Millwood)* 2010; 29: 725–731. doi: 10.1377/hlthaff.2008.0746 20368601 PMC2874878

[12] Lin SF, Beck AN, Finch BK. The Dynamic contribution of chronic conditions to temporal trends in disability among U.S. adults. *Disabil Health J* 2016; 9: 332–340. doi: 10.1016/j.dhjo.2015.11.006 26750975 PMC4808606

[13] Freedman VA, Crimmins E, Schoeni RF, et al. Old-Age Disability: Report From a Technical. 2004; 41: 417–441. 10.1353/dem.2004.0022 15461008

[14] Rijken M, Kerkhof M van, Dekker J, et al. Comorbidity of Chronic Diseases: Effects of Disease Pairs on Physical and Mental Functioning. *Qual Life Res* 2005; 14: 45–55. doi: 10.1007/s11136-004-0616-2 15789940

[15] Gijsen R, Hoeymans N, Schellevis FG, et al. Causes and consequences of comorbidity: A review. *J Clin Epidemiol* 2001; 54: 661–674. doi: 10.1016/s0895-4356(00)00363-2 11438406

[16] Fortin M, Bravo G, Hudon C, et al. Relationship between multimorbidity and health-related quality of life of patients in primary care. *Qual Life Res* 2006; 15: 83–91. doi: 10.1007/s11136-005-8661-z 16411033

[17] Tooth L, Hockey R, Byles J, et al. Weighted multimorbidity indexes predicted mortality, health service use, and health-related quality of life in older women. *J Clin Epidemiol* 2008; 61: 151–159. doi: 10.1016/j.jclinepi.2007.05.015 18177788

[18] Bayliss EA, Ellis JL, Steiner JF. Barriers to self-management and quality-of-life outcomes in seniors with multimorbidities. *Ann Fam Med* 2007; 5: 395–402. doi: 10.1370/afm.722 17893380 PMC2000313

[19] Calderón-Larrañaga A, Poblador-Plou B, González-Rubio F, et al. Multimorbidity, polypharmacy, referrals, and adverse drug events: Are we doing things well? *Br J Gen Pract* 2012; 62: 821–826. 10.3399/bjgp12X659295 PMC3505415 23211262

[20] Huntley AL, Johnson R, Purdy S, et al. Measures of multimorbidity and morbidity burden for use in primary care and community settings: A systematic review and guide. *Ann Fam Med* 2012; 10: 134–141. doi: 10.1370/afm.1363 22412005 PMC3315139

[21] Calderon-Larrañaga A, Vetrano DL, Ferrucci L, et al. Multimorbidity and functional impairment—bidirectional interplay, synergistic effects and common pathways. *J Intern Med* 2019; 285: 255–271. doi: 10.1111/joim.12843 30357990 PMC6446236

[22] Ryan A, Wallace E, O’Hara P, et al. Multimorbidity and functional decline in community-dwelling adults: a systematic review. *Health Qual Life Outcomes* 2015; 13: 1–13. 26467295 10.1186/s12955-015-0355-9 PMC4606907

[23] Hudon C, Soubhi H, Fortin M. Relationship between multimorbidity and physical activity: Secondary analysis from the Quebec health survey. *BMC Public Health* 2008; 8: 1–8. 18775074 10.1186/1471-2458-8-304 PMC2542369

[24] Prados-Torres A, Calderón-Larrañaga A, Hancco-Saavedra J, et al. Multimorbidity patterns: A systematic review. *J Clin Epidemiol* 2014; 67: 254–266. doi: 10.1016/j.jclinepi.2013.09.021 24472295

[25] Dhalwani NN, O’Donovan G, Zaccardi F, et al. Long terms trends of multimorbidity and association with physical activity in older English population. *Int J Behav Nutr Phys Act* 2016; 13: 1–9. 26785753 10.1186/s12966-016-0330-9 PMC4717631

[26] Basu S, King AC. Disability and chronic disease among older adults in India: Detecting vulnerable populations through the WHO SAGE Study. *Am J Epidemiol* 2013; 178: 1620–1628. doi: 10.1093/aje/kwt191 24049156 PMC3842902

[27] LeRoy L, Bayliss E, Domino M, et al. The agency for healthcare research and quality multiple chronic conditions research network: overview of research contributions and future priorities. *Med Care* 2014; S15–S22. doi: 10.1097/MLR.0000000000000095 24561753

[28] Depp CA, Jeste D V. Definitions and predictors of successful aging: A comprehensive review of larger quantitative studies. *Am J Geriatr Psychiatry* 2006; 14: 6–20. doi: 10.1097/01.JGP.0000192501.03069.bc 16407577

[29] Parsons S, Gale CR, Kuh D, et al. Physical capability and the advantages and disadvantages of ageing: Perceptions of older age by men and women in two British cohorts. *Ageing Soc* 2014; 34: 452–471.

[30] Kadam UT, Croft PR. Clinical multimorbidity and physical function in older adults: A record and health status linkage study in general practice. *Fam Pract* 2007; 24: 412–419. doi: 10.1093/fampra/cmm049 17698977

[31] Steeves JA, Shiroma EJ, Conger SA, et al. Physical activity patterns and multimorbidity burden of older adults with different levels of functional status: NHANES 2003–2006. *Disabil Health J* 2019; 12: 495–502. doi: 10.1016/j.dhjo.2019.02.005 30871954 PMC6629035

[32] Vancampfort D, Koyanagi A, Ward PB, et al. Chronic physical conditions, multimorbidity and physical activity across 46 low and middle income countries. *Int J Behav Nutr Phys Act* 2017; 14: 1–13. 28100238 10.1186/s12966-017-0463-5 PMC5241915

[33] Koyanagi A, Garin N, Olaya B, et al. Chronic Conditions and Sleep Problems among Adults Aged 50 years or over in Nine Countries: A Multi-Country Study. *PLOS ONE* 2014; 9: e114742. doi: 10.1371/journal.pone.0114742 25478876 PMC4257709

[34] Bertheussen GF, Romundstad PR, Landmark T, et al. Associations between physical activity and physical and mental health-A HUNT 3 study. *Med Sci Sports Exerc* 2011; 43: 1220–1228. doi: 10.1249/MSS.0b013e318206c66e 21131869

[35] Bowling CB, Deng L, Sakhuja S, et al. Prevalence of Activity Limitations and Association with Multimorbidity Among US Adults 50 to 64 Years Old. *J Gen Intern Med* 2019; 34: 2390–2396. doi: 10.1007/s11606-019-05244-8 31435766 PMC6848639

[36] Chamberlain AM, Rutten LJF, Jacobson DJ, et al. Multimorbidity, functional limitations, and outcomes: Interactions in a population-based cohort of older adults. *J Comorbidity* 2019; 9: 2235042X1987348. 10.1177/2235042X19873486 PMC6734596 31523633

[37] Sharma P, Maurya P, Muhammad T. Number of chronic conditions and associated functional limitations among older adults: cross-sectional findings from the longitudinal aging study in India. *BMC Geriatr* 2021; 21: 1–13. 34814856 10.1186/s12877-021-02620-0 PMC8609791

[38] Arokiasamy P, Uttamacharya, Jain K. Multi-morbidity, functional limitations, and self-rated health among older adults in India: cross-sectional analysis of LASI pilot survey, 2010. *Sage Open* 2015; 5: 2158244015571640.

[39] Desai S, Vanneman R. India Human Development Survey (IHDS), 2005: Version 12 [Data set]. *Inter-University Consortium for Political and Social Research*.

[40] Desai S, Vanneman R. India Human Development Survey-II (IHDS-II), 2011–12. Inter-university Consortium for Political and Social Research [distributor], 2018-08-08. 10.3886/ICPSR36151.v6.

[41] Sengupta M, Agree EM. Gender and disability among older adults in north and south India: Differences associated with coresidence and marriage. *J Cross-Cult Gerontol* 2002; 17: 313–336. doi: 10.1023/a:1023079219538 14617962

[42] Fillenbaum GG, Chandra V, Ganguli M, et al. Development of an activities of daily living scale to screen for dementia in an illiterate rural older population in India. *Age Ageing* 1999; 28: 161–168. doi: 10.1093/ageing/28.2.161 10350413

[43] Brorsson B, Asberg KH. Katz index of independence in ADL. Reliability and validity in short-term care. *Scand J Rehabil Med* 1984; 16: 125–132. 6494836

[44] Paul R, Srivastava S, Muhammad T, et al. Determinants of acquired disability and recovery from disability in Indian older adults: longitudinal influence of socio-economic and health-related factors. *BMC Geriatr* 2021; 21: 1–14. 34271879 10.1186/s12877-021-02372-x PMC8283946

[45] Srivastava S, Thalil M, Rashmi R, et al. Association of family structure with gain and loss of household headship among older adults in India: Analysis of panel data. *PLoS ONE* 2021; 16: 1–17. doi: 10.1371/journal.pone.0252722 34086833 PMC8177662

[46] Parmar MC, Saikia N. Chronic morbidity and reported disability among older persons from the India Human Development Survey. *BMC Geriatr* 2018; 18: 1–12. 30522436 10.1186/s12877-018-0979-9 PMC6284309

[47] Ram F., Mohanty SK, Ram U. Understanding the Distribution of BPL Cards: All-India and Selected States. *Econ Polit Wkly* 2009; 44: 386–408.

[48] Steinman MA, Lee SJ, Boscardin J, et al. Patterns of Multimorbidity in Elderly Veterans. *J Am Geriatr Soc* 2012; 60: 1872–1880. doi: 10.1111/j.1532-5415.2012.04158.x 23035702 PMC4133992

[49] Nagarkar A, Kashikar Y. Predictors of functional disability with focus on activities of daily living: A community based follow-up study in older adults in India. *Arch Gerontol Geriatr* 2017; 69: 151–155. doi: 10.1016/j.archger.2016.11.015 27936458

[50] Baptista FM, Rodrigues AM, Gregório MJ, et al. Functional Status and Quality of Life Determinants of a Group of Elderly People With Food Insecurity. *Front Nutr* 2018; 5: 1–8. 30410883 10.3389/fnut.2018.00099 PMC6209666

[51] Arber S, Cooper H. Gender differences in health in later life: The new paradox? *Soc Sci Med* 1999; 48: 61–76. doi: 10.1016/s0277-9536(98)00289-5 10048838

[52] Crimmins EM, Kim JK, Solé-Auró A. Gender differences in health: Results from SHARE, ELSA and HRS. *Eur J Public Health* 2011; 21: 81–91. doi: 10.1093/eurpub/ckq022 20237171 PMC3023013

[53] Muhammad T, Maurya P. Gender differences in the association between perceived income sufficiency and self-rated health among older adults: A population-based study in India. *J Women Aging* 2021; 00: 1–14. doi: 10.1080/08952841.2021.2002663 34821544

[54] Alexandre T da S, Corona LP, Nunes DP, et al. Gender differences in incidence and determinants of disability in activities of daily living among elderly individuals: SABE study. *Arch Gerontol Geriatr* 2012; 55: 431–437. doi: 10.1016/j.archger.2012.04.001 22546518

[55] Al Hazzouri AZ, Sibai AM, Chaaya M, et al. Gender differences in physical disability among older adults in underprivileged communities in Lebanon. *J Aging Health* 2011; 23: 367–382. doi: 10.1177/0898264310385454 21068395

[56] Deschênes SS, Burns RJ, Schmitz N. Associations between depression, chronic physical health conditions, and disability in a community sample: A focus on the persistence of depression. *J Affect Disord* 2015; 179: 6–13. doi: 10.1016/j.jad.2015.03.020 25841076

[57] Riebe D, Blissmer BJ, Greaney ML, et al. The relationship between obesity, physical activity, and physical function in older adults. *J Aging Health* 2009; 21: 1159–1178. doi: 10.1177/0898264309350076 19897781

[58] Quiñones AR, Markwardt S, Botoseneanu A. Multimorbidity Combinations and Disability in Older Adults. *J Gerontol—Ser Biol Sci Med Sci* 2016; 71: 823–830. doi: 10.1093/gerona/glw035 26968451 PMC4888400

[59] Whitson HE, Landerman LR, Newman AB, et al. Chronic medical conditions and the sex-based disparity in disability: The cardiovascular health study. *J Gerontol—Ser Biol Sci Med Sci* 2010; 65 A: 1325–1331. doi: 10.1093/gerona/glq139 20675619 PMC2990264

[60] Sedova B, Kalkuhl M. Who are the climate migrants and where do they go? Evidence from rural India. *World Dev* 2020; 129: 104848.

[61] Subramanian SV, Corsi DJ, Subramanyam MA, et al. Jumping the gun: The problematic discourse on socioeconomic status and cardiovascular health in India. *Int J Epidemiol* 2013; 42: 1410–1426. doi: 10.1093/ije/dyt017 23563358

[62] Prabhakaran D, Jeemon P, Roy A. Cardiovascular diseases in India: current epidemiology and future directions. *Circulation* 2016; 133: 1605–1620. doi: 10.1161/CIRCULATIONAHA.114.008729 27142605

[63] Jarvis BF. Estimating Multinomial Logit Models with Samples of Alternatives. *Sociol Methodol* 2019; 49: 341–348. doi: 10.1177/0081175018793460 33986555 PMC8114869

[64] Agborsangaya CB, Lau D, Lahtinen M, et al. Multimorbidity prevalence and patterns across socioeconomic determinants: a cross-sectional survey. *BMC Public Health* 2012; 12: 201. doi: 10.1186/1471-2458-12-201 22429338 PMC3353224

[65] Valderas JM, Starfi B, Sibbald B. Defining Comorbidity: Implications for Understanding Health and Health Services. *Ann Fam Med* 2009; 357–363. doi: 10.1370/afm.983 19597174 PMC2713155

[66] Srivastava S, Shaw S, Chaurasia H, et al. Feeling about living arrangements and associated health outcomes among older adults in India: a cross-sectional study. *BMC Public Health* 2021; 21: 1–14. 34225690 10.1186/s12889-021-11342-2 PMC8258997

[67] Banks LM, Kuper H, Polack S. Poverty and disability in low-And middleincome countries: A systematic review. *PLoS ONE* 2017; 12: 1–19. 10.1371/journal.pone.0189996 PMC5739437 29267388

[68] Audinarayana N, Sheela J. Physical disability among the elderly in Tamil Nadu: Patterns, differentials and determinants. *Health and Population*: *Perspectives and Issues* 2002; 25: 26–37.

[69] Todorov A, Kirchner C. Bias in proxies’ reports of disability: Data from the National Health Interview Survey on disability. *Am J Public Health* 2000; 90: 1248–1253. doi: 10.2105/ajph.90.8.1248 10937005 PMC1446336

